# Supplementary material for: Complexity and weak integration promote the diversity of reef fish oral jaws
Source: Commun Biol. 2024 Nov 4;7:1433. doi: 10.1038/s42003-024-07148-8 (PMC11535403; doi:10.1038/s42003-024-07148-8)
Supplement: Supplementary file 2 — Supplementary Materials [file 42003_2024_7148_MOESM2_ESM.pdf]

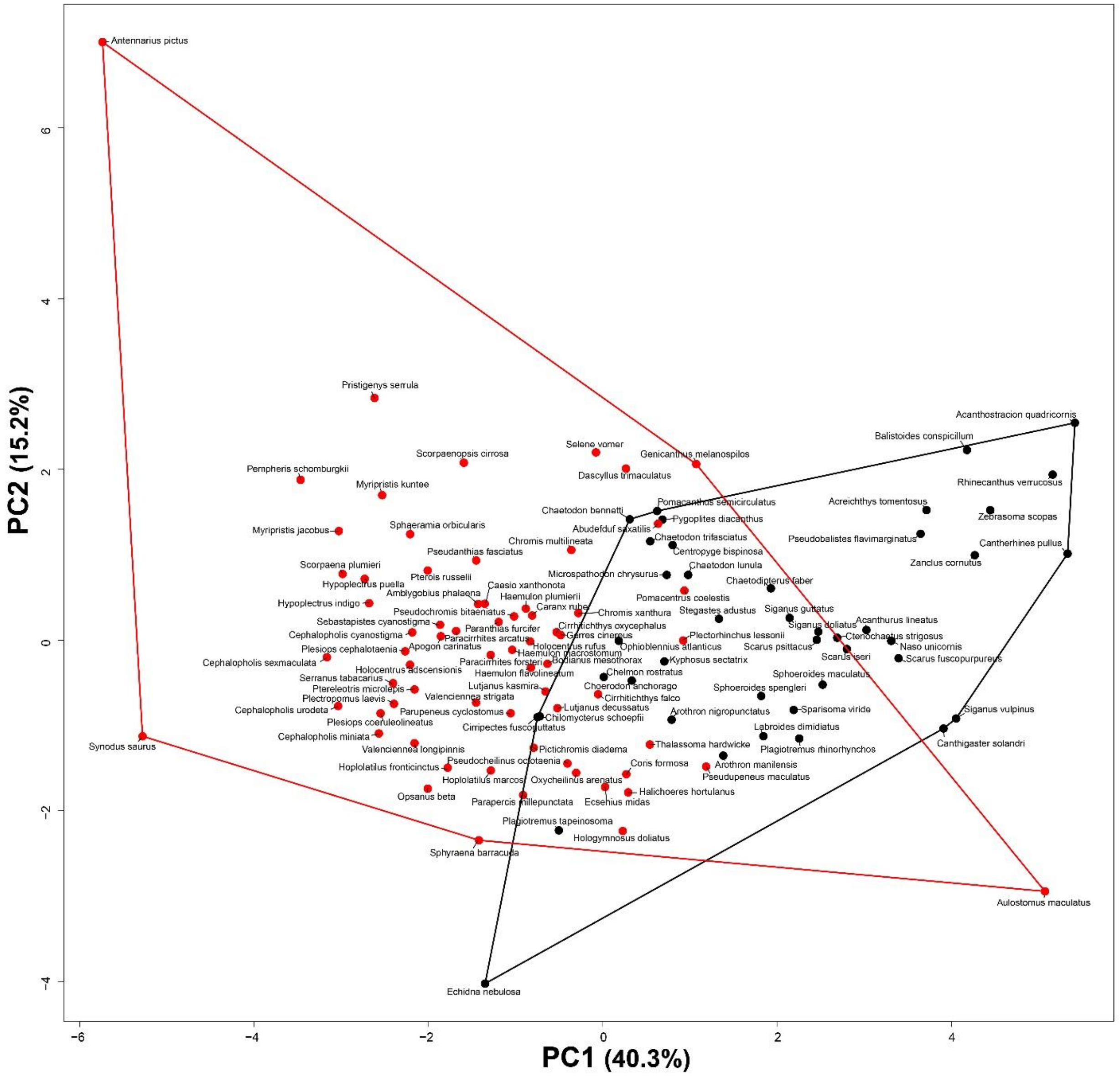

Supplementary Figure 1. Principal component analysis showing major axes of oral jaw variation in 110 species of coral reef fishes for each functional feeding mode. Each point is the average shape of a species, colored by functional feeding mode. Species names are located near each point.

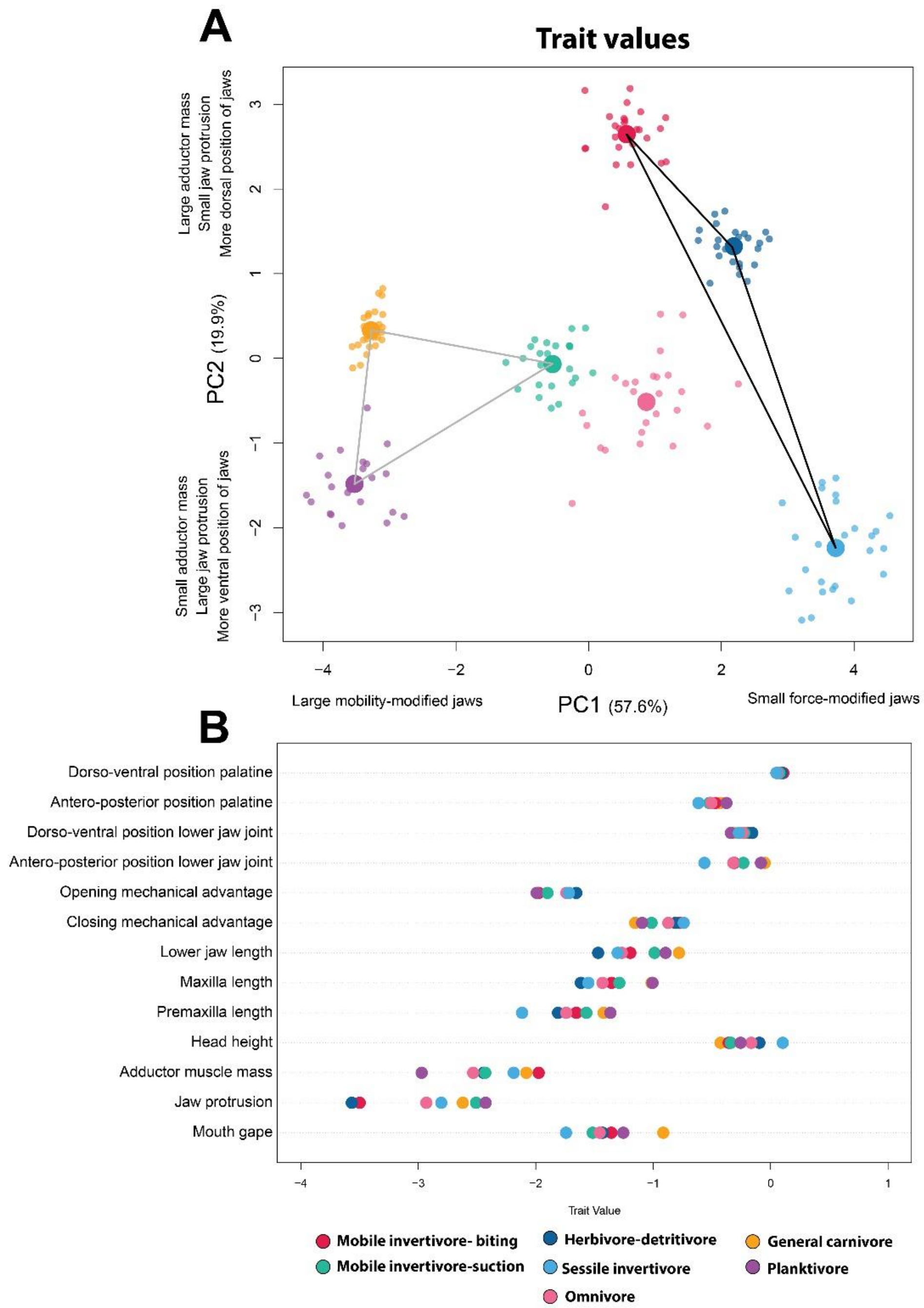

Supplementary Figure 2. (A) Principal component analysis of mean trait values for all 13 oral jaw traits showing that, while trait combinations associated with diet are largely aligned with an overarching trade-off between jaw strength and mobility, there is variation in trait combinations beyond this trend. The black polygon represents diets that feed primarily through biting. The red polygon represents diets that feed primarily through suction. Omnivores (pink circle) are represented by an almost equal split of biters and suction feeders. (B) Dotplot showing the variation in trait values between the diet groups for each trait.

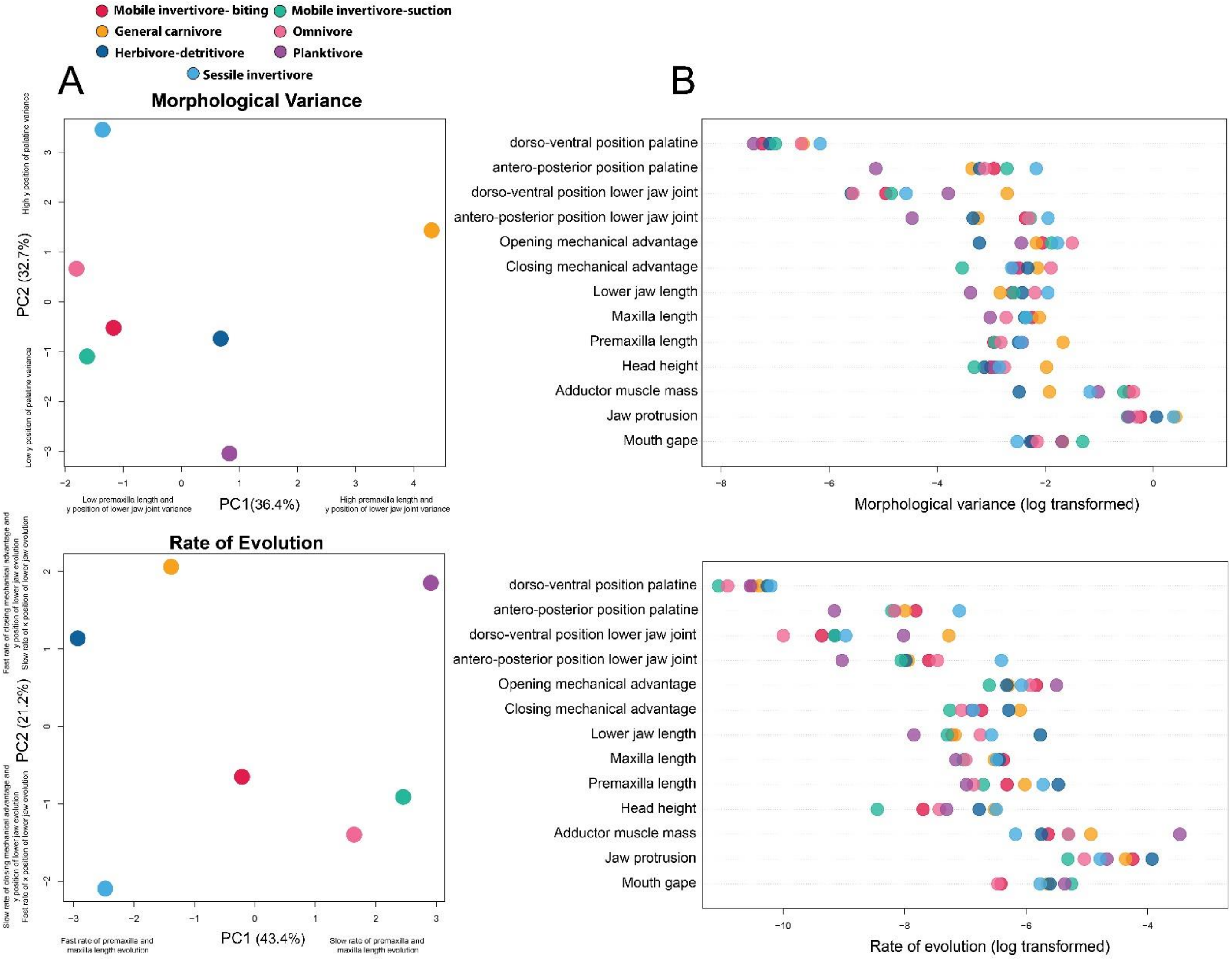

Supplementary Figure 3. (A) Principal component analyses of morphological variance and rate of evolution for oral jaw traits in each diet category. In both cases, diet reflects a variety of trait combinations. All 13 oral jaw traits were used in each Principal component analysis. (B) Dotplot showing the variation between the diet groups for each trait.

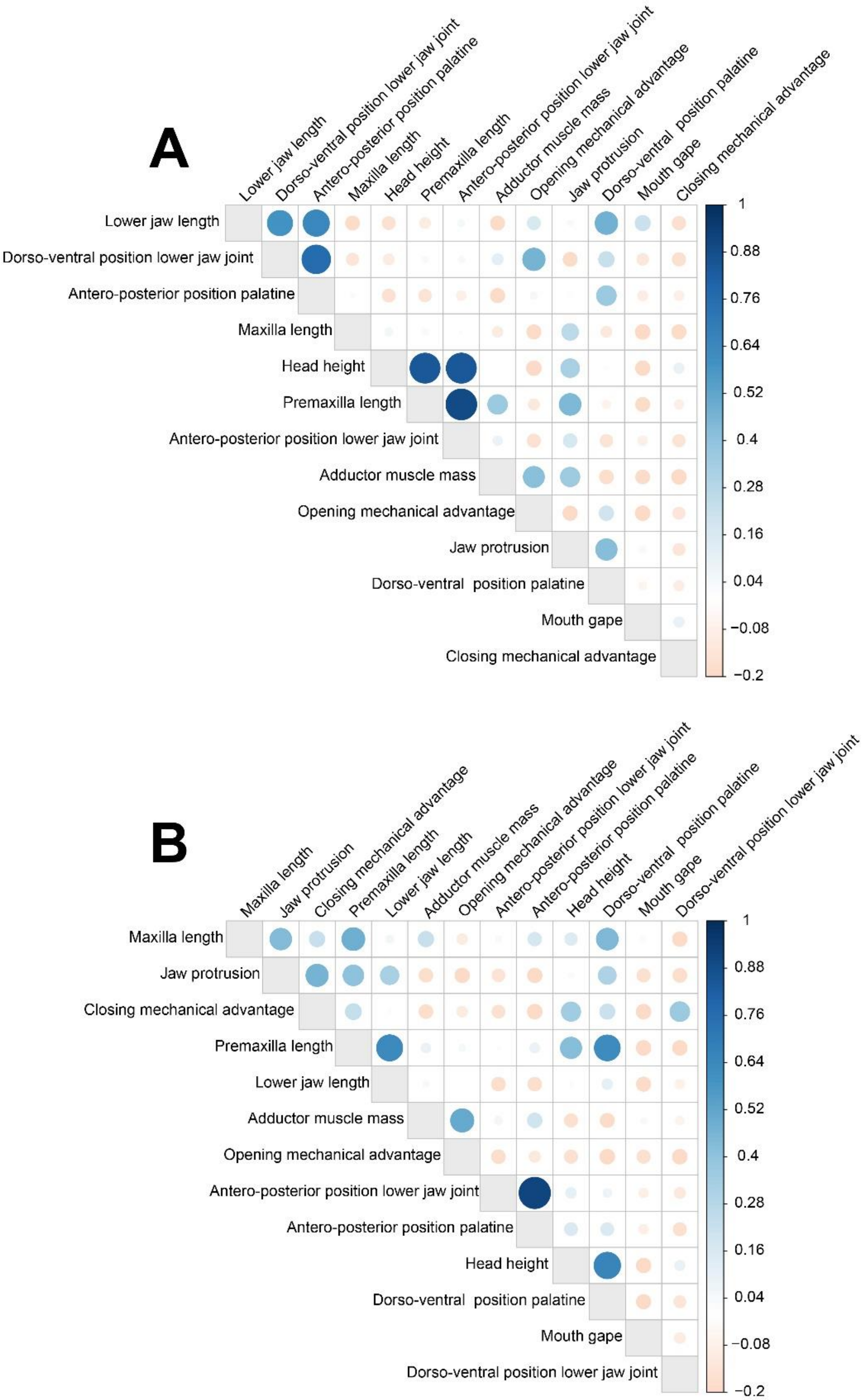

Supplementary Figure 4. Heatmap of r-squared values from the linear regression analysis of diets between the (A) morphological variance and (B) evolutionary rate values. Darker blue colors indicate a stronger positive correlation, while darker red colors indicate a stronger negative correlation. The size of the circle indicates the strength of the correlation.

Supplementary Table 1. Loadings from the principal component analysis done on species averages.

	PC1	PC2	PC3	PC4	PC5	PC6
Mouth gape	-0.30	0.08	-0.33	0.18	-0.05	0.45
Jaw protrusion	-0.22	0.29	0.38	0.14	0.30	-0.52
Adductor muscle mass	0.07	-0.10	-0.69	0.23	0.16	-0.18
Head height	0.13	0.56	-0.06	0.23	-0.09	0.01
Premaxilla length	-0.35	0.11	-0.14	0.00	0.09	-0.38
Maxilla length	-0.37	0.10	-0.27	0.07	-0.08	-0.10
Lower jaw length	-0.39	-0.05	-0.04	0.03	-0.02	-0.02
Closing mechanical advantage	0.29	0.07	-0.02	0.10	-0.78	-0.26
Opening mechanical advantage	0.17	0.47	0.14	0.34	0.23	0.40
Antero-posterior position lower jaw joint	-0.36	-0.23	0.21	0.22	-0.17	0.21
Dorso-ventral position lower jaw joint	0.25	-0.46	0.10	0.18	0.30	0.04
Antero-posterior position of anteriormost portion of palatine	-0.31	-0.17	0.31	0.29	-0.28	0.11
Dorso-ventral position of anteriormost portion of palatine	0.16	-0.21	0.00	0.74	0.05	-0.23
Proportion of variance explained	0.40	0.15	0.11	0.09	0.05	0.05
Cumulative variance explained	0.40	0.55	0.67	0.76	0.81	0.86

Supplementary Table 2. P-values from the univariate phylogenetic ANOVA, disparity, and rate analyses for each linear trait between functional feeding mode.

Trait	ANOVA	Disparity	Rate
Mouth gape	0.19	0.27	0.65
Jaw protrusion	**0.04**	0.18	0.14
Adductor muscle mass	0.33	0.94	**0.01**
Head height	0.06	0.64	0.87
Premaxilla length	**0.01**	1.00	**0.01**
Maxilla length	**0.00**	0.83	**0.02**
Lower jaw length	**0.01**	**0.00**	**0.00**
Closing mechanical advantage	**0.02**	0.42	0.90
Opening mechanical advantage	0.09	0.18	0.38
Antero-posterior position lower jaw joint	**0.01**	**0.00**	**0.01**
Dorso-ventral position lower jaw joint	**0.00**	0.44	0.51
Antero-posterior position of anteriormost portion of palatine	0.14	**0.00**	**0.02**
Dorso-ventral position of anteriormost portion of palatine	**0.02**	**0.04**	**0.02**

Supplementary Table 3. Loadings from the principal component analysis done on trait optima or means for the seven diets. *Denotes a trait in which the mean trait value was used instead of the trait optima.

	PC1	PC2	PC3	PC4
Mouth gape	-0.30	0.23	-0.08	0.04
Jaw protrusion*	-0.22	-0.44	0.06	0.16
Adductor muscle mass	0.19	0.27	-0.11	0.65
Head height*	0.30	-0.32	-0.02	-0.15
Premaxilla length	-0.37	0.14	0.03	-0.12
Maxilla length	-0.37	-0.07	0.02	0.18
Lower jaw length	0.04	0.05	0.98	0.15
Closing mechanical advantage	0.32	0.02	-0.03	0.16
Opening mechanical advantage	0.25	-0.09	0.12	-0.53
Antero-posterior position lower jaw joint*	-0.36	0.13	0.07	-0.07
Dorso-ventral position lower jaw joint*	0.25	0.43	0.03	-0.21
Antero-posterior position of anteriormost portion of palatine*	-0.32	0.14	0.01	-0.31
Dorso-ventral position of anteriormost portion of palatine	0.07	0.57	0.01	-0.09
Proportion of variance explained	0.49	0.19	0.07	0.07
Cumulative variance explained	0.49	0.68	0.75	0.83

Supplementary Table 4. Loadings from the principal component analysis done on morphological disparity for each of the seven diet categories.

	PC1	PC2	PC3	PC4
Mouth gape	0.08	-0.23	0.23	0.59
Jaw protrusion	0.25	0.39	0.14	-0.15
Adductor muscle mass	-0.34	-0.08	-0.26	0.40
Head height	0.38	0.20	-0.20	0.24
Premaxilla length	0.43	0.15	-0.02	0.10
Maxilla length	0.16	0.25	0.37	0.14
Lower jaw length	-0.25	0.38	-0.02	-0.19
Closing mechanical advantage	0.12	0.13	-0.66	-0.18
Opening mechanical advantage	-0.25	0.23	-0.34	0.43
Antero-posterior position lower jaw joint	-0.33	0.31	0.11	0.19
Dorso-ventral position lower jaw joint	0.42	0.09	-0.04	0.29
Antero-posterior position of anteriormost portion of palatine	-0.22	0.37	0.33	0.02
Dorso-ventral position of anteriormost portion of palatine	0.00	0.46	-0.10	0.08
Proportion of variance explained	0.36	0.33	0.13	0.12
Cumulative variance explained	0.36	0.69	0.82	0.94

Supplementary Table 5. Loadings from the principal component analysis done on the Brownian rate estimates for each trait in each of the seven diet categories.

	PC1	PC2	PC3	PC4
Mouth gape	0.11	0.24	0.01	-0.52
Jaw protrusion	-0.28	0.30	-0.25	0.33
Adductor muscle mass	0.25	0.34	0.31	0.19
Head height	-0.33	0.13	0.35	-0.15
Premaxilla length	-0.40	0.04	-0.10	-0.05
Maxilla length	-0.37	-0.01	-0.04	0.18
Lower jaw length	-0.29	0.01	-0.39	0.08
Closing mechanical advantage	-0.28	0.41	-0.03	-0.09
Opening mechanical advantage	0.18	0.12	0.36	0.62
Antero-posterior position lower jaw joint	-0.21	-0.42	0.37	-0.05
Dorso-ventral position lower jaw joint	-0.04	0.44	0.33	-0.30
Antero-posterior position of anteriormost portion of palatine	-0.26	-0.38	0.32	-0.10
Dorso-ventral position of anteriormost portion of palatine	-0.36	0.12	0.27	0.15
Proportion of variance explained	0.43	0.21	0.14	0.11
Cumulative variance explained	0.43	0.65	0.78	0.89

Supplementary Table 6. Not all species in our study were present in the phylogeny used in analyses. For those that were absent, we used the most closely related species in the phylogeny as a proxy. Below are the species in our data set (left column) and their replacement species (right column).*Represents spelling mistakes in the species names from the Rabosky et al. 2018 phylogeny.

Morphological Name	Tree name
Amblygobiops phalena	*Amblygobius phalaena*
Apogon novemfasciatus	*Apogon carinatus*
Assessor randalli	*Plesiops cephalotaenia*
Caesio cuning	*Caesio xanthonota*
Cephalopholis baenak	*Cephalopholis sexmaculata*
Chilomycterus schoepfi	*Chilomycterus schoepfii**
Cirrhilabrus cyanogularis	*Cirrhilabrus cyanopleura*
Crossosalarias macrospilus	*Cirripectes fuscoguttatus*
Cyprinocirrhites polyactis	*Cirrhitichthys oxycephalus*
Diodon holacanthus	*Diodon holocanthus**
Exallias brevis	*Ophioblennius atlanticus*
Gymnothorax thyrsoideus	*Gymnothorax pseudothyrsoideus*
Haemulon plumieri	*Haemulon plumierii**
Holocentrus coruscum	*Holocentrus adscensionis*
Hoplolatilus cuniculus	*Hoplolatilus fronticinctus*
Hoplolatilus fourmanoiri	*Hoplolatilus marcosi*
Labricinus cyclophthalmus	*Pseudochromis bitaeniatus*
Meiacanthus atrodorsalis	*Plagiotremus tapeinosoma*
Moncanthus hispidus	*Stephanolepis hispidus*
Oxycheilinus orientalis	*Oxycheilinus arenatus*
Parapercis tetracantha	*Parapercis millepunctata*
Paraplesiops poweri	*Plesiops coeruleolineatus*
Plectorhinchus orientalis	*Plectorhinchus lessonii*
Pomacentrus coelestris	*Pomacentrus coelestis*
Pseudanthias dispar	*Pseudanthias fasciatus*
Pterois russellii	*Pterois russelii*
Scarus fuscocaudalis	*Scarus fuscopurpureus*
Siganus guttattus	*Siganus guttatus*
Sphyraena qenie	*Sphyraena forsteri*
Taenionotus triacanthus	*Scorpaenopsis cirrosa*
Valenciennea helsdingenii	*Valenciennea longipinnis*
Zebrasoma xanthurum	*Zebrasoma scopas*

Supplementary Table 7. 110 species of coral reef fishes included in this study, with associated feeding mode and diet group designations. GC (general carnivore), MIB (mobile invertivore biters), MIS (mobile invertivore suction feeders), OM (omnivore), PK (planktivore), SI (sessile invertivore), and HD (herbivores-detritivores).

Family	Species	Feeding Mode	Diet
Acanthuridae	*Acanthurus lineatus*	biter	HD
Acanthuridae	*Ctenochaetus strigosus*	biter	HD
Acanthuridae	*Naso unicornis*	biter	HD
Acanthuridae	*Zebrasoma scopas*	biter	HD
Antennariidae	*Antennarius pictus*	suction	GC
Anthiadidae	*Pseudanthias fasciatus*	suction	PK
Apogonidae	*Sphaeramia orbicularis*	suction	MIS
Apogonidae	*Apogon carinatus*	suction	PK
Aulostomidae	*Aulostomus maculatus*	suction	GC
Balistidae	*Balistoides conspicillum*	biter	MIB
Balistidae	*Pseudobalistes flavimarginatus*	biter	MIS
Balistidae	*Rhinecanthus verrucosus*	biter	MIS
Batrachoididae	*Opsanus beta*	suction	GC
Blenniidae	*Cirripectes fuscoguttatus*	biter	HD
Blenniidae	*Ecsenius midas*	suction	PK
Blenniidae	*Plagiotremus rhinorhynchos*	biter	GC
Blenniidae	*Plagiotremus tapeinosoma*	biter	MIB
Blenniidae	*Ophioblennius atlanticus*	biter	HD
Caesionidae	*Caesio xanthonota*	suction	PK
Carangidae	*Caranx ruber*	suction	GC
Carangidae	*Selene vomer*	suction	GC
Chaetodontidae	*Chaetodon bennetti*	biter	SI
Chaetodontidae	*Chaetodon lunula*	biter	OM
Chaetodontidae	*Chaetodon trifasciatus*	biter	SI
Chaetodontidae	*Chelmon rostratus*	biter	MIS
Cirrhitidae	*Cirrhitichthys falco*	suction	GC
Cirrhitidae	*Cirrhitichthys oxycephalus*	suction	GC
Cirrhitidae	*Paracirrhites arcatus*	suction	GC
Cirrhitidae	*Paracirrhites forsteri*	suction	GC
Diodontidae	*Chilomycterus schoepfii*	biter	MIB
Ephipidae	*Chaetodipterus faber*	biter	OM
Epinephelidae	*Paranthias furcifer*	suction	PK
Epinephelidae	*Plectropomus laevis*	suction	GC
Gerreidae	*Gerres cinereus*	suction	MIS
Gobiidae	*Amblygobius phalaena*	suction	HD
Gobiidae	*Valenciennea longipinnis*	suction	OM
Gobiidae	*Valenciennea strigata*	suction	OM
Haemulidae	*Haemulon flavolineatum*	suction	MIS

Haemulidae	*Haemulon macrostomum*	suction	MIS
Haemulidae	*Haemulon plumierii*	suction	MIS
Haemulidae	*Plectorhinchus lessonii*	suction	MIS
Holocentridae	*Holocentrus adscensionis*	suction	GC
Holocentridae	*Holocentrus rufus*	suction	GC
Holocentridae	*Myripristis jacobus*	suction	PK
Holocentridae	*Myripristis kuntee*	suction	PK
Kyphosidae	*Kyphosus sectatrix*	biter	HD
Labridae	*Bodianus mesothorax*	suction	MIB
Labridae	*Choerodon anchorago*	biter	MIB
Labridae	*Coris formosa*	suction	MIB
Labridae	*Halichoeres hortulanus*	suction	MIB
Labridae	*Hologymnosus doliatus*	suction	GC
Labridae	*Labroides dimidiatus*	biter	MIB
Labridae	*Pseudocheilinus octotaenia*	suction	MIS
Labridae	*Thalassoma hardwicke*	suction	MIS
Labridae	*Oxycheilinus arenatus*	suction	GC
Lutjanidae	*Lutjanus decussatus*	suction	GC
Lutjanidae	*Lutjanus kasmira*	suction	GC
Malacanthidae	*Hoplolatilus fronticinctus*	suction	PK
Malacanthidae	*Hoplolatilus marcosi*	suction	PK
Monacanthidae	*Acreichthys tomentosus*	biter	OM
Monacanthidae	*Cantherhines pullus*	biter	SI
Mullidae	*Parupeneus cyclostomus*	suction	MIS
Mullidae	*Pseudupeneus maculatus*	suction	MIS
Muraenidae	*Echidna nebulosa*	biter	GC
Ostraciidae	*Acanthostracion quadricornis*	biter	SI
Pempheridae	*Pempheris schomburgkii*	suction	PK
Pinguipedidae	*Parapercis millepunctata*	suction	GC
Plesiopidae	*Plesiops cephalotaenia*	suction	MIS
Plesiopidae	*Plesiops coeruleolineatus*	suction	GC
Pomacanthidae	*Centropyge bispinosa*	biter	HD
Pomacanthidae	*Genicanthus melanospilos*	suction	PK
Pomacanthidae	*Pomacanthus semicirculatus*	biter	SI
Pomacanthidae	*Pygoplites diacanthus*	biter	SI
Pomacentridae	*Abudefduf saxatilis*	suction	OM
Pomacentridae	*Chromis multilineata*	suction	PK
Pomacentridae	*Chromis xanthura*	suction	PK
Pomacentridae	*Dascyllus trimaculatus*	suction	OM
Pomacentridae	*Microspathodon chrysurus*	biter	HD
Pomacentridae	*Pomacentrus coelestis*	suction	OM
Pomacentridae	*Stegastes adustus*	biter	HD
Priacanthidae	*Pristigenys serrula*	suction	GC
Pseudochromidae	*Pictichromis diadema*	suction	MIS
Pseudochromidae	*Pseudochromis bitaeniatus*	suction	GC

Ptereleotridae	*Ptereleotris microlepis*	suction	PK
Scaridae	*Sparisoma viride*	biter	HD
Scaridae	*Scarus fuscopurpureus*	biter	HD
Scaridae	*Scarus iseri*	biter	HD
Scaridae	*Scarus psittacus*	biter	HD
Scorpaenidae	*Scorpaena plumieri*	suction	GC
Scorpaenidae	*Scorpaenopsis cirrosa*	suction	GC
Scorpaenidae	*Sebastapistes cyanostigma*	suction	GC
Scorpaenidae	*Pterois russelii*	suction	GC
Serranidae	*Cephalopholis cyanostigma*	suction	GC
Serranidae	*Cephalopholis miniata*	suction	GC
Serranidae	*Cephalopholis sexmaculata*	suction	GC
Serranidae	*Cephalopholis urodeta*	suction	GC
Serranidae	*Hypoplectrus indigo*	suction	GC
Serranidae	*Hypoplectrus puella*	suction	GC
Serranidae	*Serranus tabacarius*	suction	GC
Siganidae	*Siganus doliatus*	biter	HD
Siganidae	*Siganus guttatus*	biter	HD
Siganidae	*Siganus vulpinus*	biter	HD
Sphyraenidae	*Sphyraena barracuda*	suction	GC
Synodontidae	*Synodus saurus*	suction	GC
Tetraodontidae	*Arothron manilensis*	biter	MIB
Tetraodontidae	*Arothron nigropunctatus*	biter	SI
Tetraodontidae	*Canthigaster solandri*	biter	OM
Tetraodontidae	*Sphoeroides maculatus*	biter	MIB
Tetraodontidae	*Sphoeroides spengleri*	biter	MIB
Zanclidae	*Zanclus cornutus*	biter	SI
