# Supplementary material for: Complexity and weak integration promote the diversity of reef fish oral jaws
Source: Commun Biol. 2024 Nov 4;7:1433. doi: 10.1038/s42003-024-07148-8 (PMC11535403; doi:10.1038/s42003-024-07148-8)
Supplement: Supplementary file 3 — Reporting summary [file 42003_2024_7148_MOESM3_ESM.pdf]

nature portfolio

Corresponding author(s): Michael Burns

Last updated by author(s): Oct 17, 2024

Reporting Summary

Nature Portfolio wishes to improve the reproducibility of the work that we publish. This form provides structure for consistency and transparency in reporting. For further information on Nature Portfolio policies, see our Editorial Policies and the Editorial Policy Checklist.

Statistics

For all statistical analyses, confirm that the following items are present in the figure legend, table legend, main text, or Methods section.

n/a	Confirmed	
☐	☒	The exact sample size (*n*) for each experimental group/condition, given as a discrete number and unit of measurement
☐	☒	A statement on whether measurements were taken from distinct samples or whether the same sample was measured repeatedly
☐	☒	The statistical test(s) used AND whether they are one- or two-sided<br>*Only common tests should be described solely by name; describe more complex techniques in the Methods section.*
☒	☐	A description of all covariates tested
☒	☐	A description of any assumptions or corrections, such as tests of normality and adjustment for multiple comparisons
☐	☒	A full description of the statistical parameters including central tendency (e.g. means) or other basic estimates (e.g. regression coefficient) AND variation (e.g. standard deviation) or associated estimates of uncertainty (e.g. confidence intervals)
☐	☒	For null hypothesis testing, the test statistic (e.g. *F*, *t*, *r*) with confidence intervals, effect sizes, degrees of freedom and *P* value noted<br>*Give P values as exact values whenever suitable.*
☒	☐	For Bayesian analysis, information on the choice of priors and Markov chain Monte Carlo settings
☒	☐	For hierarchical and complex designs, identification of the appropriate level for tests and full reporting of outcomes
☒	☐	Estimates of effect sizes (e.g. Cohen's *d*, Pearson's *r*), indicating how they were calculated

Our web collection on statistics for biologists contains articles on many of the points above.

Software and code

Policy information about availability of computer code

Data collection	The software Fiji version 2.35 was used to collect data on linear measurements.
Data analysis	All analysis were performed in the R statistical software version 4.2.1. The code used to perform the analysis is archived on Zenodo (https://doi.org/10.5281/zenodo.13941776).

For manuscripts utilizing custom algorithms or software that are central to the research but not yet described in published literature, software must be made available to editors and reviewers. We strongly encourage code deposition in a community repository (e.g. GitHub). See the Nature Portfolio guidelines for submitting code & software for further information.

Data

Policy information about availability of data

All manuscripts must include a data availability statement. This statement should provide the following information, where applicable:

- Accession codes, unique identifiers, or web links for publicly available datasets
- A description of any restrictions on data availability
- For clinical datasets or third party data, please ensure that the statement adheres to our policy

All data supporting the results of this study are available as Supplementary Information and archived on Figshare (https://doi.org/10.6084/m9.figshare.27245268.v1).

Research involving human participants, their data, or biological material

Policy information about studies with human participants or human data. See also policy information about sex, gender (identity/presentation), and sexual orientation and race, ethnicity and racism.

Reporting on sex and gender	N/A
Reporting on race, ethnicity, or other socially relevant groupings	N/A
Population characteristics	N/A
Recruitment	N/A
Ethics oversight	N/A

Note that full information on the approval of the study protocol must also be provided in the manuscript.

Field-specific reporting

Please select the one below that is the best fit for your research. If you are not sure, read the appropriate sections before making your selection.

☐ Life sciences ☐ Behavioural & social sciences ☒ Ecological, evolutionary & environmental sciences

For a reference copy of the document with all sections, see nature.com/documents/nr-reporting-summary-flat.pdf

Ecological, evolutionary & environmental sciences study design

All studies must disclose on these points even when the disclosure is negative.

Study description	Species included in this study are as follows: Acanthurus lineatus,Ctenochaetus strigosus, Naso unicornis, Zebrasoma scopas, Antennarius pictus, Pseudanthias fasciatus, Sphaeramia orbicularis, Apogon carinatus, Aulostomus maculatus, Balistoides conspicillum, Pseudobalistes flavimarginatus, Rhinecanthus verrucosus, Opsanus beta, Cirripectes fuscoguttatus, Ecsenius midas, Plagiotremus rhinorhynchos, Plagiotremus tapeinosoma, Ophioblennius atlanticus, Caesio xanthonota, Caranx ruber, Selene vomer, Chaetodon bennetti, Chaetodon lunula, Chaetodon trifasciatus, Chelmon rostratus, Cirrhitichthys falco, Cirrhitichthys oxycephalus, Paracirrhites arcatus, Paracirrhites forsteri, Chilomycterus schoepfii, Chaetodipterus faber, Paranthias furcifer, Plectropomus laevis, Gerres cinereus, Amblygobius phalaena, Valenciennea longipinnis, Valenciennea strigata, Haemulon flavolineatum, Haemulon macrostomum, Haemulon plumierii, Plectorhinchus lessonii, Holocentrus adscensionis, Holocentrus rufus, Myripristis jacobus, Myripristis kuntee, Kyphosus sectatrix, Bodianus mesothorax, Choerodon anchorago, Coris formosa, Halichoeres hortulanus, Hologymnosus doliatus, Labroides dimidiatus, Pseudocheilinus octotaenia, Thalassoma hardwicke, Oxycheilinus arenatus, Lutjanus decussatus, Lutjanus kasmira, Hoplolatilus fronticinctus, Hoplolatilus marcosi, Acreichthys tomentosus, Cantherhines pullus, Parupeneus cyclostomus, Pseudupeneus maculatus, Echidna nebulosa, Acanthostracion quadricornis, Pempheris schomburgkii, Parapercis millepunctata, Plesiops cephalotaenia, Plesiops coeruleolineatus, Centropyge bispinosa, Genicanthus melanospilos, Pomacanthus semicirculatus, Pygoplites diacanthus, Abudefduf saxatilis, Chromis multilineata, Chromis xanthura, Dascyllus trimaculatus, Microspathodon chrysurus, Pomacentrus coelestis, Stegastes adustus, Pristigenys serrula, Pictichromis diadema, Pseudochromis bitaeniatus, Ptereleotris microlepis, Sparisoma viride, Scarus fuscopurpureus, Scarus iseri, Scarus psittacus, Scorpaena plumieri, Scorpaenopsis cirrosa, Sebastapistes cyanostigma, Pterois russelii, Cephalopholis cyanostigma, Cephalopholis miniata, Cephalopholis sexmaculata, Cephalopholis urodeta, Hypoplectrus indigo, Hypoplectrus puella, Serranus tabacarius, Siganus doliatus, Siganus guttatus, Siganus vulpinus, Sphyraena barracuda, Synodus saurus, Arothron manilensis, Arothron nigropunctatus, Canthigaster solandri, Sphoeroides maculatus, Sphoeroides spengleri, Zanclus cornutus
Research sample	We collected linear measurements on 110 species of coral reef fishes.
Sampling strategy	We sampled species from all the major reef fishes to capture the broad evolutionary history of the group.
Data collection	Lateral photographs of specimens were taken by all authors. Linear measurements were calculated on photographs using the software Fiji. All authors collected linear measurements.
Timing and spatial scale	The data was collected on preserved specimens.
Data exclusions	No data were excluded from this study.
Reproducibility	All analyses were run repeatedly using the code supplied on Zenodo (https://doi.org/10.5281/zenodo.13941776).
Randomization	We used permutations or simulations to randomize the data during significance testing.
Blinding	Species names and ecological state were withheld during linear measurements to reduce bias.

Did the study involve field work? ☐ Yes ☒ No

Reporting for specific materials, systems and methods

We require information from authors about some types of materials, experimental systems and methods used in many studies. Here, indicate whether each material, system or method listed is relevant to your study. If you are not sure if a list item applies to your research, read the appropriate section before selecting a response.

Materials & experimental systems

n/a	Involved in the study
☒	☐ Antibodies
☒	☐ Eukaryotic cell lines
☒	☐ Palaeontology and archaeology
☐	☒ Animals and other organisms
☒	☐ Clinical data
☒	☐ Dual use research of concern
☒	☐ Plants

Methods

n/a	Involved in the study
☒	☐ ChIP-seq
☒	☐ Flow cytometry
☒	☐ MRI-based neuroimaging

Animals and other research organisms

Policy information about studies involving animals; ARRIVE guidelines recommended for reporting animal research, and Sex and Gender in Research

Laboratory animals	Species included in this study are as follows: Acanthurus lineatus,Ctenochaetus strigosus, Naso unicornis, Zebrasoma scopas, Antennarius pictus, Pseudanthias fasciatus, Sphaeramia orbicularis, Apogon carinatus, Aulostomus maculatus, Balistoides conspicillum, Pseudobalistes flavimarginatus, Rhinecanthus verrucosus, Opsanus beta, Cirripectes fuscoguttatus, Ecsenius midas, Plagiotremus rhinorhynchos, Plagiotremus tapeinosoma, Ophioblennius atlanticus, Caesio xanthonota, Caranx ruber, Selene vomer, Chaetodon bennetti, Chaetodon lunula, Chaetodon trifasciatus, Chelmon rostratus, Cirrhitichthys falco, Cirrhitichthys oxycephalus, Paracirrhites arcatus, Paracirrhites forsteri, Chilomycterus schoepfii, Chaetodipterus faber, Paranthias furcifer, Plectropomus laevis, Gerres cinereus, Amblygobius phalaena, Valenciennea longipinnis, Valenciennea strigata, Haemulon flavolineatum, Haemulon macrostomum, Haemulon plumierii, Plectorhinchus lessonii, Holocentrus adscensionis, Holocentrus rufus, Myripristis jacobus, Myripristis kuntee, Kyphosus sectatrix, Bodianus mesothorax, Choerodon anchorago, Coris formosa, Halichoeres hortulanus, Hologymnosus doliatus, Labroides dimidiatus, Pseudocheilinus octotaenia, Thalassoma hardwicke, Oxycheilinus arenatus, Lutjanus decussatus, Lutjanus kasmira, Hoplolatilus fronticinctus, Hoplolatilus marcosi, Acreichthys tomentosus, Cantherhines pullus, Parupeneus cyclostomus, Pseudupeneus maculatus, Echidna nebulosa, Acanthostracion quadricornis, Pempheris schomburgkii, Parapercis millepunctata, Plesiops cephalotaenia, Plesiops coeruleolineatus, Centropyge bispinosa, Genicanthus melanospilos, Pomacanthus semicirculatus, Pygoplites diacanthus, Abudefduf saxatilis, Chromis multilineata, Chromis xanthura, Dascyllus trimaculatus, Microspathodon chrysurus, Pomacentrus coelestis, Stegastes adustus, Pristigenys serrula, Pictichromis diadema, Pseudochromis bitaeniatus, Ptereleotris microlepis, Sparisoma viride, Scarus fuscopurpureus, Scarus iseri, Scarus psittacus, Scorpaena plumieri, Scorpaenopsis cirrosa, Sebastapistes cyanostigma, Pterois russelii, Cephalopholis cyanostigma, Cephalopholis miniata, Cephalopholis sexmaculata, Cephalopholis urodeta, Hypoplectrus indigo, Hypoplectrus puella, Serranus tabacarius, Siganus doliatus, Siganus guttatus, Siganus vulpinus, Sphyraena barracuda, Synodus saurus, Arothron manilensis, Arothron nigropunctatus, Canthigaster solandri, Sphoeroides maculatus, Sphoeroides spengleri, Zanclus cornutus
Wild animals	N/A
Reporting on sex	Sex was not determined for each species.
Field-collected samples	N/A
Ethics oversight	This research was conducted in accordance with the University of California, Davis' Institutional Animal Care and Use Committee (protocol #22206)

Note that full information on the approval of the study protocol must also be provided in the manuscript.

Plants

Seed stocks	N/A
Novel plant genotypes	N/A
Authentication	N/A
